# Modeling Sequential Dependencies in Progressive Matrices: An Auto-Regressive Item Response Theory (AR-IRT) Approach

**DOI:** 10.3390/jintelligence12010007

**Published:** 2024-01-15

**Authors:** Nils Myszkowski, Martin Storme

**Affiliations:** 1 Department of Psychology, Pace University, New York, NY 10004, USA; 2 IESEG School of Management, Univ. Lille, CNRS, UMR 9221 - LEM - Lille Économie Management, 59000 Lille, France

**Keywords:** psychometrics, progressive matrices, item response theory, local dependencies, test motivation

## Abstract

Measurement models traditionally make the assumption that item responses are independent from one another, conditional upon the common factor. They typically explore for violations of this assumption using various methods, but rarely do they account for the possibility that an item predicts the next. Extending the development of auto-regressive models in the context of personality and judgment tests, we propose to extend binary item response models—using, as an example, the 2-parameter logistic (2PL) model—to include auto-regressive sequential dependencies. We motivate such models and illustrate them in the context of a publicly available progressive matrices dataset. We find an auto-regressive lag-1 2PL model to outperform a traditional 2PL model in fit as well as to provide more conservative discrimination parameters and standard errors. We conclude that sequential effects are likely overlooked in the context of cognitive ability testing in general and progressive matrices tests in particular. We discuss extensions, notably models with multiple lag effects and variable lag effects.

## 1. Introduction

Progressive matrices tests stand at the forefront of cognitive assessment, serving as a cornerstone in the study of fluid intelligence, a critical component of cognitive functioning. The intricate design of these tests, which progressively increase in complexity, necessitates the application of sophisticated psychometric models to ensure precise measurement and interpretation. While the psychometric properties of progressive matrices have been extensively explored in multiple ways—including in a Special Issue of this very journal (Myszkowski 2021)—there remains a notable gap in the literature: the lack of consideration for sequential dependencies. Although psychometric research has studied sequential effects in personality tests (Tang et al. 2020), traditional psychometric approaches to modeling progressive matrices have largely overlooked the potential influence of item sequences on test performance. This oversight may obscure the nuanced dynamics of test-taking behavior, where the response to a given item could be contingent upon the outcomes of preceding items. As such, there is a pressing need to integrate sequential dependencies into psychometric models to capture the full spectrum of cognitive processes elicited by progressive matrices tests.

Building upon recent advances in psychometric modeling and the development of sophisticated statistical software, this paper proposes to integrate sequential dependencies into the psychometric investigation of progressive matrices. Recognizing the intricate interplay between successive test items, and based on recently proposed threshold auto-regressive item response theory (TAR-IRT) models used to model dependencies in polytomous personality questionnaires, we propose an auto-regressive item response theory (AR-IRT) model, which, as an extension of binary response models, accounts for these dependencies, offering a more granular and accurate depiction of test-taker performance. In doing so, we aim to bridge the gap identified in the literature and enrich the psychometric analysis of progressive matrices with a more comprehensive understanding of test dynamics. The remainder of this introduction will lay the groundwork for our study, beginning with a review of the historical context and significance of progressive matrices. Subsequent sections will discuss the evolution of psychometric models in intelligence measurement, the limitations of current approaches, and the theoretical underpinnings of sequential dependencies. Finally, we will articulate the rationale behind our study, outline our objectives, and provide a preview of the paper’s structure.

### 1.1. Significance of Progressive Matrices Tests

Progressive matrices are a family of psychometric tests that are extensively recognized within the field of intelligence research—they probably require little by way of introduction to the readers of this journal. Originally developed by Raven (1941), these tests present a series of patterns with a missing piece, tasking the test-taker with identifying the correct piece from a set of options. The matrices are ’progressive’ in the sense that they increase in difficulty with each subsequent item. The items are designed to measure deductive reasoning and non-verbal abstract problem-solving abilities—hallmarks of fluid intelligence (Carpenter et al. 1990).

Over the decades, they have been refined and adapted, yielding several versions to suit a range of age groups and cognitive abilities (Bors and Stokes 1998; Langener et al. 2022; Myszkowski and Storme 2018; Raven 2000). Their enduring relevance is reflected in the vast array of studies that have employed these matrices to explore the dimensions of human intelligence (Kaufman et al. 2009), as well as in their continued use in educational, occupational, and clinical settings. Moreover, the utility of Raven’s Progressive Matrices transcends the realm of human cognitive assessment, extending into the domain of artificial intelligence where they pose a formidable challenge in the quest to develop a model capable of human-like reasoning and problem-solving (Kunda 2020).

In addition to their foundational role in intelligence research, Raven’s Progressive Matrices serve a practical purpose in the broader psychological assessment landscape. The original matrices and their revisions and shortened versions are frequently employed as a succinct measure of general intelligence (Myszkowski and Storme 2018), offering a less time-consuming alternative to more extensive test batteries. Although sacrificing depth of insight into an individual’s cognitive capabilities (Gignac 2015), this efficiency makes them particularly valuable in large-scale studies or in contexts where testing time is at a premium. Their ability to provide a quick yet robust gauge of g-factor, or general intelligence, is one of the many reasons they remain a staple in both research and applied settings.

### 1.2. Psychometric Modeling of Progressive Matrices

The measurement of intelligence via Raven’s Progressive Matrices has been approached using various psychometric models, each offering unique insights into the underlying cognitive processes. Here, we will present an overview of the plethora of methods that have been used.

Classical Test Theory (CTT) has provided foundational measures, such as Cronbach’s α Cronbach (1951) to assess internal consistency and sum scores to represent overall performance. Although CTT methods—congeneric models aside—present the advantage of permitting the calculation of person scores (via sum/average scoring as proxies for “true scores”) without requiring the estimation of a psychometric model, this added simplicity often oversimplifies the functional relation between latent attributes and item responses (Borsboom 2006; McNeish and Wolf 2020), especially in the context of binary responses. Still, in the context of progressive matrices, sum/average scoring can provide reasonably good proxies for more accurate (but also more complex) scoring methods based on latent variable models (Myszkowski and Storme 2018).

Non-parametric scaling, such as Mokken scale analysis (Mokken 1971; Mokken and Lewis 1982; Van der Ark 2007), offers an alternative framework to study the psychometric properties of tests. It allows us to study the properties of items and tests without assuming a parametric form for the item response function (Mokken 1971). Such non-parametric response functions notably allow us to study some anticipated features of progressive matrices tests, such as the monotonicity of the response function (the assumption that an item response function should be monotonously increasing at all levels of the latent trait). Non-parametric scaling, via Mokken scale analysis, has been applied to Raven’s matrices to assess the item response function monotonicity and the hierarchical ordering of items (Myszkowski 2020b).

Item Response Theory (IRT) has become a cornerstone in the psychometric analysis of cognitive ability tests in general, and of Raven’s matrices in particular, with models ranging from the one-parameter logistic (1PL) model to the more complex two-parameter (2PL) and three-parameter (3PL) models, and even the four-parameter logistic (4PL) model (Bürkner 2020; Myszkowski and Storme 2018). These models offer a probabilistic framework that (notably) accounts for the difficulty and discrimination parameters of each item, as well as for guessing and slipping phenomena, allowing for a more accurate estimation of test-taker abilities. We may add that item response models have been fitted on progressive matrices tests using a variety of modeling and estimation frameworks. For example, IRT models fitted using a Bayesian Generalized Linear Multilevel Modeling (GLMM) framework have been used Bürkner (2020), with packages such as brms (Bürkner 2017). Generalized Structural Equation Modeling (SEM) frameworks have also been used to fit IRT models as categorical confirmatory factor analysis models (Myszkowski and Storme 2018) with packages such as lavaan (Rosseel 2012). And, of course, full-information item response theory frameworks, which utilize all available response patterns to estimate model parameters, have also been used to fit item response models on Raven’s matrices tests (Myszkowski and Storme 2018), notably using the mirt package (Chalmers 2012).

Although progressive matrices item responses are generally analyzed as pass–fail binary responses and distractor responses, and how their information can be integrated in person-ability estimation has also been studied in the context of progressive matrices. For categorical data that extend beyond binary responses, models such as the nominal response model (Bock 1972) and the nested logit model (Suh and Bolt 2010) provide a framework for analyzing responses with multiple categories, capturing the full complexity of item responses in person ability estimation. These methods have been discussed as ways to increase the reliability of person estimates—especially at low ability levels—in the context of progressive matrices (Myszkowski and Storme 2018; Storme et al. 2019). Statistical methods for detecting distractor responses that could have discrimination power have also been discussed in the context of progressive matrices (Forthmann et al. 2020).

Recent psychometric advances have also seen the development (and rediscovery) of modern methods for dimensionality assessment, such as Exploratory Graph Analysis (Golino and Epskamp 2017; Golino et al. 2020) and exploratory bifactor models (Reise et al. 2010). These methods have also been used in the study of responses to progressive matrices tests Garcia-Garzon et al. (2019) and have provided further insight into their structure, and the extent to which they can be assumed—as they usually are—to be unidimensional.

Each of these psychometric approaches provides a unique lens through which the complexity of Raven’s Progressive Matrices can be examined. The choice of model often reflects a balance between the theoretical considerations of the test’s construct and the practical constraints of data and computational resources, as well as they can also be used to address a particular gap in the literature. In the following section, we will discuss one gap that has not yet been addressed.

### 1.3. Local Dependencies and Their Violations

In both Classical Test Theory (CTT) and Item Response Theory (IRT), a fundamental assumption is that of local independence. This principle posits that, given a person’s level on the latent attribute being measured (e.g., intelligence), their responses to individual test items are statistically independent of one another. In other words, the response to one item does not affect the probability of a certain response to another item. Violations of this assumption, known as local dependencies (or simply, violations of local independence), occur when responses to items are statistically related, over and beyond what can be accounted for by the latent trait. There can be several reasons for the occurrence of local dependencies in cognitive ability tests. For example, in speeded tests, final items may be counted as failed items when examinees have not reached them, and as a consequence, failure to reach an item implies failing subsequent items (Chen and Thissen 1997). It is also possible that some items include similar content (e.g., vocabulary items from the same semantic domain), which is essentially a problem of the test not being unidimensional for some items (Chen and Thissen 1997). Another possibility is that an alternative strategy may be available to examinees when solving a subset of the items of a test. More directly relevant to this particular study, one possible explanation for local dependencies is that “examinees may learn while taking tests“ (Chen and Wang 2007, p. 388)—as a consequence, their success to an item (which indicates their successful learning of a rule) may condition, above and beyond their cognitive ability, their success towards the next items.

Recognizing and accounting for local dependencies in a test is crucial, as their presence can lead to biased parameter estimates and compromised test validity. A number of studies have focused on how the failure to account for such effects impacts the estimation of person (and item) estimates (Chen and Wang 2007). When local dependencies are present and unaccounted for, the likelihood function, which that describes the probability of the observed patterns of responses across items, becomes incorrectly specified as the product of the individual probabilities of the item responses. This misrepresentation can lead to distorted estimates of both the test-taker’s ability (person parameters) and the properties of the test items themselves (item parameters), and items involved in local dependencies provide less information about the latent attribute than the model assumes. As a consequence, failure to account for local dependencies ultimately compromises the validity of the test (Chen and Wang 2007; Chen and Thissen 1997).

In psychometric practice, the detection of violations of local independence is typically an empirical endeavor, where statistical techniques are employed post hoc to identify unexpected item correlations (e.g., Chen and Wang 2007), such as local dependency measures (in IRT) and modification indices (in CFA). However, this reactive approach can sometimes lead to model over-fitting, where the model becomes excessively complex in an attempt to account for these dependencies, potentially capturing noise rather than true systematic patterns. A more proactive strategy involves conceptual forethought about the characteristics of the test that may give rise to dependencies. By anticipating the types of item interrelations that could logically occur—such as those stemming from the content, structure, or sequencing of questions—we can tailor our models to accommodate these dependencies from the outset. This forward-thinking approach not only helps in constructing more robust psychometric models, but also aids in preserving the parsimony of the model, ensuring that it remains generalizable and reflective of the construct it aims to measure.

### 1.4. Sequential Local Dependencies

Sequential local dependencies—or simply sequential dependencies—in psychometric assessments refer to the influence of a preceding item’s response on the subsequent item(s) (Annis and Malmberg 2013; Shimada and Katahira 2023). This is often conceptualized as a ‘lag effect’, where the outcome of one item carries over to impact the response to subsequent items (Shimada and Katahira 2023; Tang et al. 2020). When the lag effect is positive (i.e., the expected response to an item is increased with the previous response), we may refer to it as a positive sequential dependency (Annis and Malmberg 2013). Sequential dependencies have been studied in the context of personality questionnaires (Shimada and Katahira 2023; Tang et al. 2020), notably using models that include auto-regressive parameters—added to IRT (Tang et al. 2020) or multilevel models (Shimada and Katahira 2023)—in which the probability of a response to an item is partially predicted by the response to a preceding item, in addition to the latent attribute.

Sequential dependencies are particularly pertinent in the context of personality questionnaires because accounting for them allows us to capture and account for various psychological effects that occur in personality testing situations. For example, Ozkok et al. (2019) propose that, in personality tests, memory activation and affective priming may cause sequential dependencies. Memory activation in survey responses can be understood as a process where answering questions about a particular construct sequentially taps into the same associative networks, leading to a primed state that facilitates retrieval for subsequent items. Affective priming in surveys suggests that the mood elicited by responding to an item can influence subsequent responses, creating serial dependencies that are not directly related to the construct being measured, but rather, to the affective context established by previous items.

In contrast, sequential dependencies in cognitive ability tests—and, perhaps even more surprisingly, in progressive tests—remain largely undiscussed. There are however reasons, both empirical and conceptual, to suspect that progressive matrices tests in particular may be subject to sequential dependencies. First, a recent study (Garcia-Garzon et al. 2019) on a shortened progressive matrices test indicated the possibility of a nuisance factor for the last half of the test. The fact that the nuisance factor seems to concern items that are consecutive may lead us to suspect that what has been detected in an exploratory manner may in fact better be described as a consequence of sequential dependency effects.

On a more substantive level, it has been previously advanced that learning during a test may cause sequential dependencies (Chen and Wang 2007). Indeed, tests like progressive matrices are built so that succeeding an item does not only depend on a person’s cognitive ability, but also on their successful solving of previous items. This is particularly true of items right before a given item because they would involve rules and combinations of rules that are similar. This implies that, given a person’s cognitive ability level, when a person succeeds an item, they are better positioned to succeed the next because they have successfully discerned patterns that can be reused or extended. This phenomenon could manifest statistically as a positive sequential dependency, where succeeding an item increases the probability to succeed the next, given a person’s ability.

Emotional and motivational responses to test items can also contribute to sequential dependencies. The experience of solving an item correctly can provide a self-efficacy boost, enhancing confidence and cognitive efficiency for the next item. Conversely, failure to solve an item may lead to a temporary drop in self-efficacy (Bandura 1977), as the respondent receives a feedback that their ability does not match the level of difficulty of the task, which may affect confidence and performance on subsequent items. These emotional and motivational dynamics are critical to understanding the performance trajectory during an assessment.

Attentional fluctuations during test taking are another source of sequential dependencies. The cognitive demand of progressive matrices may lead to variations in focus, with attention potentially waning or intensifying based on the perceived difficulty or engagement level of the items. For example, it has been suggested, via response time IRT models, that individuals, even in non-speeded tests, tend to vary in speed (and thus possibly effort), during a progressive test—some individuals remain relatively stable, while some increase in speed (they are more and more hasty) as others slow down (they are more and more cautious and usually perform better than the other groups) (Myszkowski et al. 2022). Such fluctuations of motivation and effort can result in the success of an item depending on the success of a previous item, over and beyond the examinee’s ability level.

Given these cognitive, emotional, and attentional factors, it is plausible to posit that sequential dependencies are a realistic aspect of the test-taking experience in progressive matrices. We would argue that the progressive nature of these tests, designed to increase in difficulty and to introduce rules that are reused in subsequent items, naturally lends itself to the occurrence of such dependencies. Therefore, recognizing and modeling these dependencies is not only a methodological advancement but also a step towards a more nuanced understanding of the test-taking process itself.

## 2. Auto-Regressive IRT Models

In order to account for sequential dependencies within a test, auto-regressive terms may be added to a measurement model. We will first briefly discuss here the 2-parameter logistic model, and then extend it to auto-regressive models.

### 2.1. The 2-Parameter Logistic Model

Given a set of persons i=1,2,…,N and items j=1,2,…,p, the probability of a correct response to item *j* by person *i* in a 2-parameter logistic (2PL) model is modeled as:

(1) $$P\left(X_{ij}=1|\theta_{i}\right)=\frac{1}{1+\exp(-a_{j}\left(\theta_{i}-b_{j}\right))}.$$

In this equation, $X_{ij}$ is the response of person *i* to item *j*, $\theta_{i}$ is the latent trait (ability) of person *i*, $a_{j}$ is the discrimination parameter of item *j*, and $b_{j}$ is the difficulty parameter of item *j*. As can be seen, item responses are not predicted by any other item response, which illustrates the assumption of conditional independence.

#### 2.1.1. Auto-Regressive 2-Parameter Logistic Model with Lag 1

From the 2PL model, we can define an auto-regressive IRT model with a lag of 1 item (AR1-IRT), which adds a sequential dependency. We therefore have an auto-regressive 2PL model with lag 1 (AR1-2PL), whose item response function can be defined for all items after the first (for which a 2PL response model is used) as

(2) $$P\left(X_{ij}=1|\theta_{i},X_{i,j-1}\right)=\frac{1}{1+\exp(-a_{j}\left(\theta_{i}-b_{j}\right)-lX_{i,j-1})}.$$

**Figure 1:**
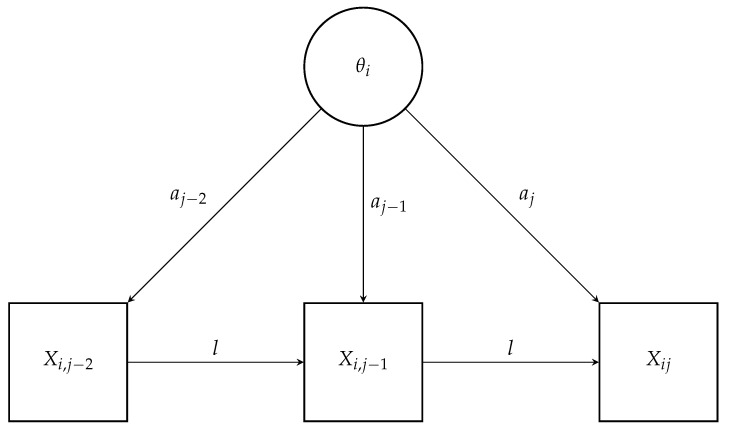
Conceptual representation of an auto-regressive IRT model with (fixed) lag 1.

In this AR1-2PL model, $X_{ij}$ represents the response of person *i* to item *j*, where $\theta_{i}$ denotes the latent trait of the individual, $a_{j}$ is the item discrimination parameter, $b_{j}$ is the item difficulty parameter, and *l* represents the auto-regressive parameter for the lag 1 effect, capturing the influence of the preceding item response $X_{i,j-1}$ on the current response. The model is conceptually represented in Figure 1.

We can note here that the lag effect (captured with the *l* parameter) is assumed constant across the items. Alternatively, we may define an AR1-2PL model with variable lag, in which each item may differ in strength (and direction) of its sequential dependency:

(3) $$P\left(X_{ij}=1|\theta_{i},l_{j-1}\right)=\frac{1}{1+\exp(-a_{j}\left(\theta_{i}-b_{j}\right)+l_{j}X_{i,j-1})}.$$

In this AR1-2PL model with variable lag, $X_{ij}$ represents the response of person *i* to item *j*, $\theta_{i}$ denotes the latent trait of the individual, $a_{j}$ is the item discrimination parameter, $b_{j}$ is the item difficulty parameter, and $l_{j}$ denotes the auto-regressive parameter for the lag 1 effect.

#### 2.1.2. Auto-Regressive 2-Parameter Logistic Model with *k* Lags

From the AR1-2PL, we can define more general models that can accommodate more lags. We obtain a more general formula for an auto-regressive 2PL model with *k* lags (AR*k*-2PL):

(4) $$P\left(X_{ij}=1|\theta_{i},X_{i,j-1},X_{i,j-2},\ldots ,X_{i,j-k}\right)=\frac{1}{1+\exp\left(-a_{j}\left(\theta_{i}-b_{j}\right)-\sum_{m=1}^{k}l_{m}X_{i,j-m}\right)}.$$

**Figure 2:**
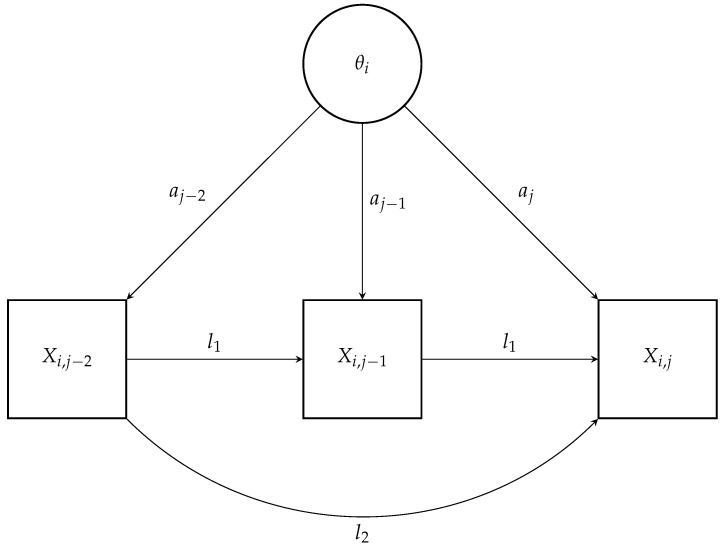
Conceptual representation of an auto-regressive IRT model with (fixed) lag 1 and 2.

In this model with *k* lags (k=1,2,…,p), $\theta_{i}$ denotes the latent trait of the individual, $a_{j}$ is the item discrimination parameter, $b_{j}$ is the item difficulty parameter, and $l_{m}$ represents the auto-regressive parameter for the lag *m* effect, capturing the influence of the item response *m* steps back on the current item response. The sum $\sum_{m=1}^{k}l_{m}X_{i,j-m}$ represents the total effect of the previous *k* item responses on the current item response. The model is conceptually represented for k=2 (lags 1 and 2) in Figure 2. In a similar manner to previously, the auto-regressive model with *k* lags can be extended to accommodate variable lag effects across items, replacing fixed lag parameters $l_{m}$ with item specific lag parameters $l_{jm}$:

(5) $$P\left(X_{ij}=1|\theta_{i},X_{i,j-1},X_{i,j-2},\ldots ,X_{i,j-k}\right)=\frac{1}{1+\exp\left(-a_{j}\left(\theta_{i}-b_{j}\right)-\sum_{m=1}^{k}l_{jm}X_{i,j-m}\right)}.$$

The auto-regressive 2PL models presented here may be seen as particular cases of the more general threshold auto-regressive IRT (TAR-IRT) models (Tang et al. 2020) used for ordinal responses. However, they can be extended to other response models than the 2PL model (e.g., 3PL models), which is here used for its popularity and for illustrative purposes.

### 2.2. Model Selection Considerations

#### 2.2.1. Choosing between Fixed and Variable Lag Parameters

To note, the number of lag parameters to be estimated in a model with *k* fixed lag effects is simply *k*, but, in a variable lag model, it can be calculated as $\sum_{j=1}^{p}\min\left(j-1,k\right)$. Consequently, although variable lag models are more flexible, they can quickly come at the cost of being heavily parameterized (and item response models already tend to require large sample sizes for calibration). Probably for this reason (although not explicitly discussed), to the best of our knowledge, auto-regressive models for sequential dependencies are regularly presented with lag effects that are fixed across items rather than variable (Tang et al. 2020).

In addition, the relative simplicity of fixed lag models allows for clearer interpretation of the results. This is because, in the context of a fixed lag model, if a positive dependency effect is observed, it suggests an overall effect of sequential dependency across the test, which is directly interpretable as the sign of an overall sequential phenomenon occurring in the test (e.g., from learning). Inversely, a variable lag model represents a multiple testing situation, where spurious sequential effects might appear while others might not, which is probably hardly interpretable in most contexts. To note, these are general approaches, but an alternative that may be relevant in some contexts is to specify and test specific lag effects in what we could perhaps describe as an auto-regressive IRT model with sparse lags. For instance, an item that requires the recall of previously learned information may prime the respondent for similar items that follow, whereas other items may not trigger such a recall. However, modeling sparse lag effects might be unrealistic in most psychometric situations and would require the careful consideration of which specific lags to include in the model.

#### 2.2.2. Selecting a Number of Lags (*k*)

In the specific context of progressive matrices, including lag-1 effects (and, to a lesser extent lag-2 effects) in the model is particularly relevant. Progressive matrices typically involve a series of problems where each problem requires the identification and application of a rule or pattern. The immediate (lag-1) effect is crucial here, as the understanding or solution of one problem often directly influences the approach or solution to the next. This is because the respondent is likely to apply a recently learned rule or pattern in the immediate next item. The lag-2 effect, while less direct, can still be relevant in cases where the respondent needs an additional item to fully grasp or apply a learned rule. Beyond lag-2, the effects are likely to become increasingly diffuse and less directly attributable to specific prior responses. Thus, in progressive matrices, the most relevant sequential effects are likely to be observed in the immediate or near-immediate responses following an item. Consequently, although in theory more lag effects could be included, we would recommend that in most contexts, lag effects beyond a lag-2 should not be considered, with the exception of situations where an item is specifically designed as a tutorial item for a series of subsequent items.

### 2.3. Information

In IRT, the information function plays a critical role in understanding the utility of test items, as it quantifies how much information an item provides about an examinee’s ability level. The concept of item information is fundamentally linked to the squared derivative of the probability function with respect to the ability parameter $\theta_{i}$. For a standard 2-Parameter Logistic (2PL) model, the information function $I(\theta_{i})$, for a given item at a specific ability level $\theta_{i}$ is defined as:

(6) $$I\left(\theta_{i}\right)=a_{j}^{2}\times P\left(\theta_{i}\right)\times \left(1-P\left(\theta_{i}\right)\right),$$

where $a_{j}$ is the discrimination parameter of the item, and $P(\theta_{i})$ is the probability of a correct response at ability level $\theta_{i}$. When extending the 2PL model to include a lag-1 effect, the probability function evolves to $P(\theta_{i}|X_{j-1})$, thereby incorporating the influence of the previous item response, $X_{j-1}$. While the lag-1 effect alters the probability of success, it is treated as a constant in the differentiation process with respect to $\theta_{i}$: This is because the lag-1 effect, although affecting the response probability, does not interact with the $\theta_{i}$ parameter in the model’s formulation. Therefore, the essential nature of the derivative with respect to $\theta_{i}$, which is central to calculating item information, remains intact. Consequently, the information function in this extended model simply differs from that of the standard 2PL model by a different (and conditional upon $X_{j-1}$) calculation of $P(\theta_{i}|X_{j-1})$ (which is the item response function):

(7) $$I\left(\theta_{i}|X_{i,j-1}\right)=a_{j}^{2}\times P\left(\theta_{i}|X_{j-1}\right)\times \left(1-P\left(\theta_{i}|X_{j-1}\right)\right).$$

This modification implies that the item’s information is now dynamically influenced by the sequential pattern of responses. For a lag-1 effect, this would imply that all items (except the first item) have two information functions, one for whether they succeeded the previous item, one if they did not. We may refer then to these functions as conditional item information functions.

### 2.4. Objectives and Hypothesis

In the empirical part of this paper, we aimed at illustrating the use of an auto-regressive binary IRT model in the context of progressive matrices. The primary goal of this empirical study is to demonstrate in a dataset (where sequential effects could be suspected) that an auto-regressive IRT model can outperform a non-auto-regressive IRT model, and that using an auto-regressive model results in differences, especially in person estimates and their standard errors.

We hypothesized that

1. an auto-regressive IRT model would outperform a non-auto-regressive model in model fit;
2. a non-negligible positive lag-1 effect would be observed, indicating that successfully solving an item increases the probability of correctly responding to the next item (over and beyond the effect of the common factor);
3. standard errors of person estimates would be larger in the auto-regressive model, indicating that using a traditional IRT (i.e., non-auto-regressive)—in cases where an auto-regressive approach is relevant conceptually (like we argued) and empirically (like we hypothesized)—results in overestimating test information/reliability.

Apart from this set of hypotheses, we also performed a number of additional explorations. These additional explorations are discussed in Section 3.

## 3. Methods

### 3.1. Dataset

To demonstrate the use of sequential models in the context of progressive matrices tests, we reanalyzed a publicly available dataset with 499 responses from adults to the last (and most difficult) series of the standard progressive matrices (SPM–LS; Myszkowski and Storme 2018). The SPM–LS is a non-verbal matrices test, comprised of 12 items of increasing difficulty. This dataset has been extensively discussed and reanalyzed in a Special Issue of this very journal (Myszkowski 2020a) using a plethora of approaches. Since a number of properties of the test have already have been presented, we will here only mention, because it is relevant, that the responses were coded as binary pass–fail.

### 3.2. Models Estimated

The original analysis of the dataset suggested that 2-, 3- and 4-parameter logistic models provided a good fit for the responses to the test. Due to its popularity, availability in various software, and the fact that it allowed to augment a relatively parsimonious model, we used, as baseline (i.e., local independence model), the 2-parameter logistic model discussed previously.

### 3.3. Model Parameterization and Estimation

All models were estimated using the commercial software Mplus (Muthén and Muthén 1998). Although we think that other packages, either based on the IRT framework, the generalized multilevel linear modeling (GLMM) framework, or the generalized SEM framework, could be capable of estimating some or all of the estimated models, Mplus was chosen over other packages for its speed of estimation, ease of use, ability to fit 2PL models, and its ability to include auto-regressive terms that are both fixed and variable. In this software, the lag-1 effects are essentially specified as predictors in regression paths.

All models were estimated using the maximum likelihood estimator, with 10,000 iterations, and converged successfully. The MplusAutomation package (Hallquist and Wiley 2018) was used to then extract parameters from the estimated models and further analyze them in R.

In Mplus, IRT logistic models are estimated using a slope-intercept parameterization. In the 2PL model, the probability of a correct response to an item is modeled as a logistic function of the latent trait or ability ($\theta_{i}$) and item parameters. Specifically, the probability of a correct response (*P*) by individual *i* to item *j* is expressed as

(8) $$P\left(X_{ij}=1|\theta_{i}\right)=\frac{1}{1+\exp(-\left(-\tau_{j}+\alpha_{j}\theta_{i}\right))}=\frac{1}{1+\exp(\tau_{j}-\alpha_{j}\theta_{i})},$$

where $\tau_{j}$ represents the item’s threshold (difficulty parameter) and $\alpha_{j}$ is the item’s slope (discrimination parameter). This parameterization effectively captures the relationship between an individual’s ability and the likelihood of a correct response, with the threshold acting as a negative intercept in the logistic model.

When extending this model to include a lag effect, as in the case of a fixed lag-1 model, the formulation incorporates the response to the previous item ($X_{i,j-1}$). The extended model is thus parameterized as

(9) $$P\left(X_{ij}=1|\theta_{i},X_{i,j-1}\right)=\frac{1}{1+\exp(-\left(-\tau_{j}+\alpha_{j}\theta_{i}+\beta_{j}X_{i,j-1}\right))}=\frac{1}{1+\exp(\tau_{j}-\alpha_{j}\theta_{i}-\beta_{j}X_{i,j-1}))}.$$

In this model, $\beta_{j}$ represents the lag parameter, quantifying the influence of the previous item’s response on the current item’s response. A positive value of $\beta_{j}$ (lag-1) indicates a positive sequential dependency, suggesting that a correct response on the previous item increases the probability of a correct response on the current item.

### 3.4. Model Comparisons and Interpretations

All model comparisons were performed using likelihood ratio tests, computed from the Mplus log-likelihood of the different models. To consider model parsimony beyond null hypothesis significance testing, we also used Akaike information criteria (AIC) and the Bayesian information criteria (BIC) provided directly in Mplus.

We interpreted the direction of the lag effect using the lag parameter estimates $\hat{\beta_{j}}$. Although asymptotically equivalent with the likelihood ratio test, we also reported the Wald *z* test of these estimates. We also converted the lag parameter estimate to an odds ratio metric (which is the most common metric used to assess effect size in logistic models) by exponentiating it (i.e., $exp(\hat{\beta_{j}})$). For further interpretation, we computed and plotted item response functions and item information functions—both conditional upon $X_{i,j-1}$—for all items in the auto-regressive model.

Expected a posteriori (EAP) $\theta_{i}$ estimates and their standard errors were extracted directly from Mplus. We compared standard errors using relative efficiency (RE), computed as the ratio of the measurement variances (i.e., a between-models ratio of the squared standard errors). We compared $\theta_{i}$ estimates between models using average bias (average pairwise difference), and root mean squared difference (RMSD) between estimates. For inference, all of these comparison statistics were bootstrapped (with 5000 resamples) and bias corrected, and accelerated (BCa) confidence intervals were computed using the R package boot.

### 3.5. Additional Explorations

We performed additional analyses using different models to further explore the possibilities of auto-regressive binary IRT models in progressive matrices test contexts. First, we estimated a model with variable lag-1 effects to investigate the variability of lag effects between items. Through a nested model comparison between the variable lag-1 and the fixed lag-1 model, we essentially tested whether lag-1 effects significantly differed across items. Although we consider that it is in general more parsimonious to assume that lag effects are fixed (and also more useful in the perspective of quantifying and testing such an effect), we anticipated that lag-1 effects might differ between items, and that most (if not all) would be positive.

In addition, we estimated a model with both (fixed) lag-1 and lag-2 effects (AR2-2PL), to test if the item response $X_{i,j-2}$ before the previous item had an effect on the probability to succeed item $X_{i,j}$, over and beyond the effect of the previous item $X_{i,j-1}$ and over and beyond the common factor $\theta_{i}$. Although we did not have a strong hypothesis regarding the existence of such an effect, we speculated that, if any, the effect would be positive (passing $X_{i,j-2}$ would increase the probability to pass $X_{i,j}$), but that the effect would remain of smaller magnitude than the lag-1 effect (i.e., there would be an auto-correlation decay) because it was more remote.

## 4. Results

### 4.1. Fixed Lag-1 Effect

As hypothesized (Hypothesis 1), the fixed lag-1 auto-regressive 2PL model significantly outperformed the (non-auto-regressive) 2PL model, both considering the likelihood ratio test and information criteria—$\chi^{2}\left(1\right)=28.122,p<$ .001, $AIC_{2\mathrm{PL}}=5631.556,AIC_{\mathrm{AR}1-2\mathrm{PL}}=5605.433,BIC_{2\mathrm{PL}}=5732.659,BIC_{\mathrm{AR}1-2\mathrm{PL}}=5710.748$.

From the estimated models, we simulated datasets with the original sample size (with person locations drawn from a standard normal distribution), using the item response functions of the 2PL and of the AR1-2PL model. For each model, 1000 datasets were simulated. Likelihood ratio tests (with a threshold of .05) incorrectly rejected the 2PL model in 6.00% of the datasets where the 2PL model was correct (5.90% for the *z* test of the lag effect). Likelihood ratio tests correctly rejected the 2PL model in favor of the AR1-2PL in all of the datasets where the AR1-2PL model was correct (it was the same proportion for the *z* test of the lag effect). In this context, it thus appears that model estimation in Mplus and likelihood ratio tests were able to provide a correct model comparison in the present context.

Item parameters of both models are reported in Table 1. Between the 2PL and the AR1-2PL model, the item difficulties were nearly perfectly correlated—r(10) = .995, p< .001—while the difficulties of the AR1-2PL model (M=−1.091) were higher than those of the 2PL model (M=−1.531)—t(11)=5.91, p< .001. The item discriminations were also nearly perfectly correlated—r(10)= .992,p< .001—while the discriminations of the AR1-2PL model (M=1.835) were lower than those of the 2PL model (M=2.101)—t(11)=24.47, p< .001. Interestingly, between-model differences in difficulties and discriminations were themselves strongly negatively correlated—r(10)=−.753,p= .005.

**Figure 3:**
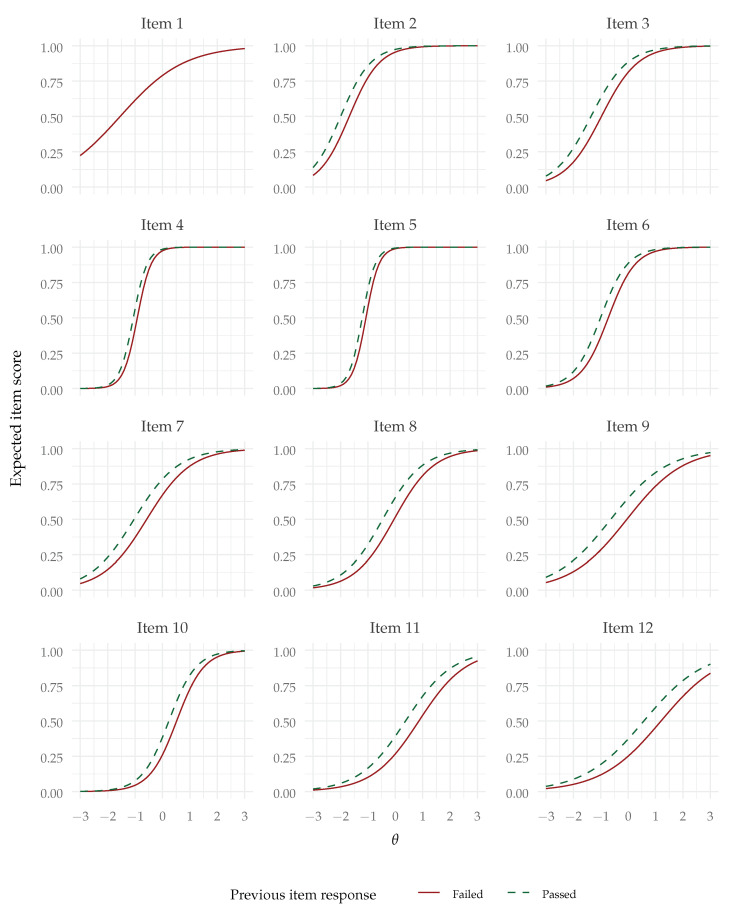
Item response functions for the AR1-2PL model (note: Item 1 has no lag effect).

**Figure 4:**
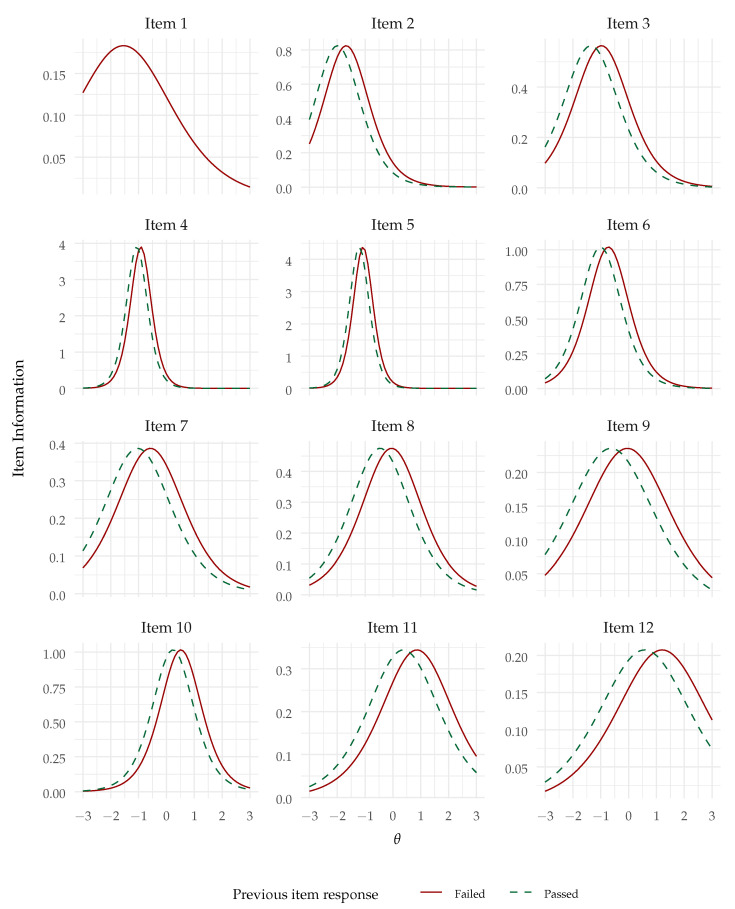
Information functions for the AR1-2PL model (note: Item 1 has no lag effect).

Also as hypothesized (Hypothesis 2), the lag-1 effect was significantly positive—B=0.572,SE=0.107,z=5.321,p<.001. The odds ratio (controlling for the effect of the common factor) was 1.771, indicating that the odds of succeeding an item were predicted to be multiplied by 1.771 after having passed the previous item, compared with having failed it. Although there are no commonly accepted standards for odds ratios in this context, odds ratios above 1.5 like this one are in general regarded as indicative of small (but non-negligible) effects. The lag effect can be visualized in the conditional item response functions plot in Figure 3. We can see that the lag effect might be regarded as a shift in difficulty from succeeding or passing the previous item. Since the item response functions are now conditional upon the previous item response (except for item 1), the item information functions, presented in Figure 4, are also conditional upon the previous item response and present a similar shift.

**Figure 5:**
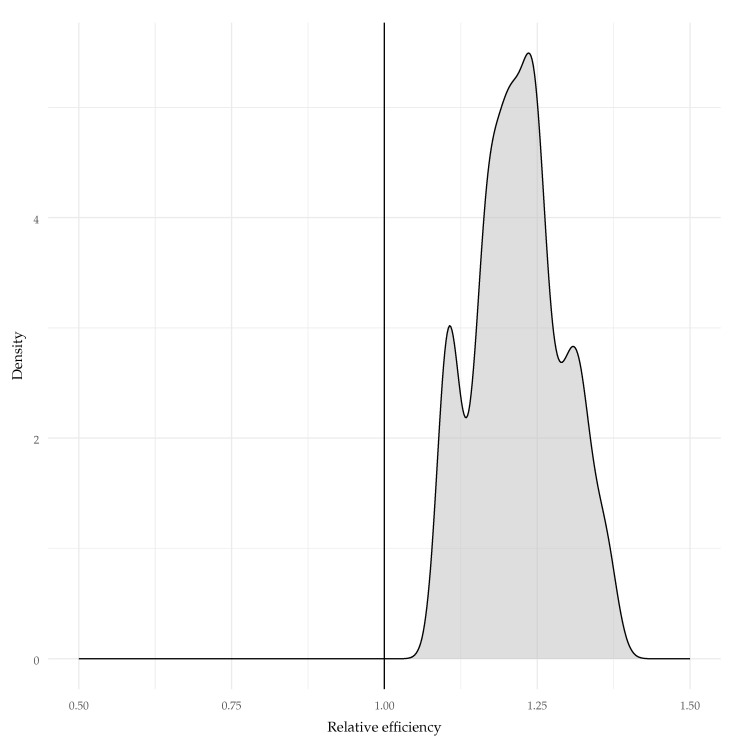
Relative efficiency of the AR1-2PL model vs. 2PL in the precision of person parameters (values greater than one indicate that the 2PL has smaller standard errors).

As hypothesized (Hypothesis 3), differences in standard errors of person estimates were observed between the models. Controlling for lag effects resulted in larger standard errors for the auto-regressive lag-1 model than for the standard 2PL model. The mean relative efficiency across all cases was 1.219, which indicates that the estimation variance of $\theta_{i}$ was about 22% larger in the lag-1 model. The bootstrapped confidence intervals of the relative efficiency excluded 1—$BCI_{95\%}\left[1.189,1.203\right]$—indicating a significant difference. This indicates that the reliability estimated for the standard 2PL model is significantly larger than the one estimated for the AR1-2PL model. In other words, if we assume that the AR1-2PL is a correct model, then the 2PL significantly overestimated test reliability. A density plot of the relative efficiencies across all cases is provided in Figure 5.

No significant bias (i.e., no systematic error) was found when comparing person estimates across the two models ($M_{\mathrm{Bias}(\theta_{i})}=0.003,BCI_{95\%}\left[-0.001,0.007\right]$). The person estimates were significantly different but minimal, as their root mean squared difference was 0.051 ($BCI_{95\%}\left[0.049,0.054\right]$), which corresponds to typical differences of about 0.05 standard deviations. This indicates that, overall, the person estimates were very close across the two models.

### 4.2. Additional Analyses

#### 4.2.1. Variable Lag Model

In the investigation of the variability between items in lag effects, we found that an AR1-2PL with variable lag outperformed in fit the AR1-2PL, with a fixed lag per the likelihood ratio test and AIC, but not BIC—$\chi^{2}\left(10\right)=57.886,p<$ .$001,AIC_{\mathrm{Fixed}}=5605.433$, $AIC_{\mathrm{Variable}}=5567.549$, $BIC_{\mathrm{Fixed}}=5710.748,BIC_{\mathrm{Variable}}=5714.990$. Balancing this mixed finding with the possible instability resulting from freeing 10 additional parameters, we considered it was more cautious to conclude that the fixed model was, in this case, a more optimal choice for interpretation.

**Figure 6:**
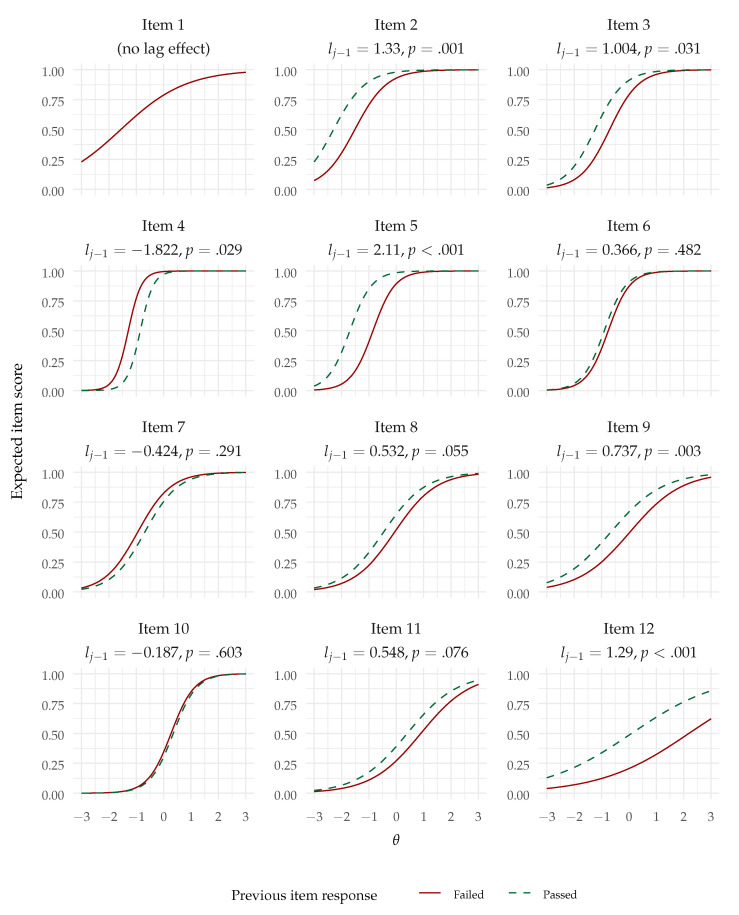
Item response functions for the AR1-2PL model with variable lag (note: Item 1 has no lag effect).

Still, we can note that, in the variable lag model, out of 11 lag effects, eight lag effects were positive (predicting responses to items 2, 3, 5, 6, 8, 9, 11, and 12), out of which five were significantly positive at p< .05 and two were marginally significantly positive at p< .10. Only one lag effect (predicting item 4) was significantly negative at p< .05. The estimates and their *p* values are reported with the conditional item response functions in Figure 6. Overall, this result indicates that sequential dependencies were mostly positive in the variable lag model.

#### 4.2.2. Lag-2 model

The comparison of the (fixed) AR1-2PL with a model that also included lag-2 effects (AR2-2PL) indicated that there was a significant lag-2 effect—$\chi^{2}\left(1\right)=9.646$, p< .001, $AIC_{\mathrm{AR}1-2\mathrm{PL}}=5605.433$, $AIC_{\mathrm{AR}2-2\mathrm{PL}}=5597.788$, $BIC_{\mathrm{AR}1-2\mathrm{PL}}=5710.748$, $BIC_{\mathrm{AR}2-2\mathrm{PL}}=5707.316$.

Like in the AR1-2PL, in the AR2-2PL model, the lag-1 effect was significantly positive—B=0.667,SE=0.116,z=5.771,p< .001. The odds ratio for the lag-1 effect was 1.948, indicating that the odds of succeeding an item were predicted to be multiplied by 1.948 when having passed the previous item, compared with having failed it.

In this model, as we had speculated, the lag-2 effect was significantly positive—B=0.370,SE=0.121,z=3.051,p= .002—and smaller than the lag-1 effect, indicating an auto-correlation decay. The odds ratio for the lag-2 effect was 1.448, indicating that the odds of succeeding an item were predicted to be multiplied by 1.448 when having passed the item before the previous item, compared with having failed it. In spite of the significance of this effect, an odds ratio of this magnitude is generally regarded as below the “small” threshold, so we would argue that the AR1-2PL remains a more parsimonious account of the sequential dependencies in this dataset.

## 5. Discussion

### 5.1. Summary of Findings

The assumption of local independence is central to the accurate calibration (and therefore interpretation) of measurement models. In the common practice of item response theory modeling, local dependencies are typically viewed as possible nuisance relations that must be explored in a dataset, so that they could be resolved—for example, by removing problematic items. Yet, in the context of a number of psychometric tests, it can be argued that some local dependencies are, in fact, structurally present because of foreseeable psychological phenomena (e.g., rater effects). While a number of local dependencies can be well accounted for using additional latent or manifest causal variables (e.g., nuisance/specific factors), there are also situations where item responses can be influenced by previous responses directly. The dependencies of item responses on the next may be referred to as sequential dependencies (Annis and Malmberg 2013; Ozkok et al. 2019; Tang et al. 2020).

Sequential dependencies have not been extensively studied in item response modeling, and when they have, they have been studied in the context of accounting for response biases occurring in personality tests (Ozkok et al. 2019; Tang et al. 2020) and judgment tests (Annis and Malmberg 2013), such as halo effects. For these situations, the models proposed have been extensions of confirmatory factor analysis and polytomous IRT models to include auto-regression effects.

Here, we propose that sequential dependencies may be suspected in a number of performance tests, and notably, tests that are built with the goal of examinees learning rules through solving items that are later applied in subsequent items, such as progressive matrices. We thus propose extensions of traditional binary IRT models, taking the 2PL model as an example, to include auto-regressive effects. After discussing various extensions (multiple lag models and variable lag models), we illustrated the use of an auto-regressive 2PL model in a publicly available progressive matrices dataset.

Our analysis showed that, in this example dataset, as we hypothesized, the auto-regressive 2PL model outperformed the non-auto-regressive 2PL model. Further, we found that the lag-1 effects were significantly positive, implying that succeeding an item increases the probability to pass the next, over and beyond one’s latent ability. The investigation of the person estimates indicated no significant bias in the person estimates from using a non-auto-regressive model (and significant but minimal differences in the estimates overall), but the investigation of the standard errors indicated that not accounting for sequential dependencies led to significantly overestimating reliability. These results indicate that (1) accounting for sequential dependencies in this example data resulted in an improvement in model fit, and that (2) doing so led to similar person estimates but less optimistic standard errors.

Item parameter comparisons between the 2PL and AR1-2PL models indicated that, although the parameter estimates were nearly perfectly correlated across models, they differed on average, with items being more difficult and less discriminant per the AR1-2PL model. This indicates that failing to account for sequential dependencies could lead to overestimating discrimination and underestimating difficulty. Furthermore, larger differences in difficulty were associated with larger differences in discrimination. We may speculate that this result could be due to general interactions between difficulty and discrimination in the two models in this dataset, as the correlation between intercepts and discriminations was −.783 in the 2PL model and −0.771 in the AR1-2PL model, which is in line with the correlation observed of −.753 in parameter differences across models. Alternatively, we may speculate that this result implies that items that are the most impacted in their difficulty by accounting for sequential effects are also the most impacted in discrimination in general because these items share common features (e.g., they may engage processes that are more affected by sequential effects). Replications in other datasets are warranted to further investigate whether this is a feature of the dataset analyzed here, a feature of progressive matrices, and/or a feature of the models tested.

Our further investigations did not clarify whether a variable lag-1 model was preferable in terms of fit, and we therefore recommend to study variable lag models only if conceptually reasonable. In this particular context, we concluded that it was more parsimonious and sufficient to account for overall dependencies, but it still appeared that there was some between-item variability in the lag-1 effects that were observed, even though most were significantly positive.

Finally, we investigated an additional lag effect (AR2-2PL) and found that its fit outperformed the model with only one lag. However, we noted that, in this model, the lag-1 effect was actually slightly similar in magnitude than in the AR1-2PL model, and that the lag-2 effect that was added, although significantly positive, was smaller in magnitude than the lag-1 effect and could be considered negligible when looking at the corresponding odds ratio.

### 5.2. Limitations and Future Studies

Although we think that the use of sequential IRT models can be interesting in a number of testing contexts, the present study presents a number of limitations that may be addressable in future research. We will try to enumerate a few of them.

First, we concentrated our efforts here on the study of one dataset. It is clear that future research on the topic of sequential IRT models should investigate a larger number of datasets. Analyzing new datasets could allow us to better understand the extent to which the effects found here can be extended to progressive matrices tests in general and perhaps to other types of performance tests as well. Although we believe that progressive matrices tests, because of their design, are ideal candidates for the illustration of sequential IRT models, there may be other tests in which sequential effects may be suspected and found, and others where they may not.

Additionally, in this paper, we focused on motivating, defining, and illustrating sequential binary IRT models. As a consequence, additional analyses may be feasible but were “out of focus”. For example, we focused on the 2-parameter logistic model as baseline here, but sequential models could be explored using other response models—binary or not. We also used only one statistical estimation software, and other studies may focus on the implementation of sequential models in various frameworks and software. For example, the implementation of sequential binary logistic models in open-source packages like mirt (Chalmers 2012), lavaan (Rosseel 2012), PLMixed (Jeon and Rockwood 2018), or brms (Bürkner 2017) seems possible, although different software may impose different constraints, notably in the possibility to allow for variable lag effects in calibration feasibility or in the possibility to use more parametrized response models than the 2PL.

Future studies may also focus on how the magnitude of lag effects might be moderated by a number of situational or dispositional variables. It could be speculated, for example, that an individual’s perseverance, grit, and/or internal locus of control would tend to de-correlate one’s success at an item from the previous one because one may be better able to regroup when they fail an item. Oppositely, one’s emotional instability or sensitivity to feedback may increase the magnitude of sequential dependencies. In general, the models proposed here consider lag effects to be fixed across persons (whether they are fixed or not across items). The models may be extended in future studies to random lag effects, which would better account for (as well as allow us to explain) between-person variability in susceptibility to sequential effects. Regarding situational explanatory variables, we may also speculate that low stakes testing situations may lead to more sequential phenomena, such as giving up (Myszkowski et al. 2022), which could also be a cause of sequential dependencies. Further, giving immediate feedback on item success or failure may also reinforce sequential dependencies.

Although this dataset does not include explanatory variables that could be used as item covariates, there may be identifiable characteristics of certain items that could explain why sequential effects may be smaller or larger (or possibly in opposite directions). Notably, from our qualitative observations, we shall note that items 1 through 3 are solved through adding layers of visual patterns, while the next items imply subtractions of layers. This may explain why the lag effect on item 4 is the only significantly negative lag effect. We would speculate that the addition strategy is primed in the first three items (if solved successively), and that an examinee who succeeded item 3 may be more strongly primed and less likely to switch strategy for the next item and succeed it. This difficulty to switch previously primed cognitive strategies is often referred to as an Einstellung effect (Luchins 1942). In line with this hypothesis, the strongest (positive) lag that was observed is on the next item (item 5), which also tends to suggest that item 4 has a pivotal role in the test, a point where the examinee has to inhibit a previously successful strategy, and to mobilize their cognitive flexibility in order to successfully change strategies and solve the next items.

The auto-regressive models delineated herein, while discussed within the framework of binary logistic IRT models, are not confined to binary response models alone. They could be extended to encompass a broader spectrum of models tailored for other types of response data. For instance, count responses, response times, or visual analog scale responses, which are encountered in a number of psychological and educational assessments, could potentially benefit from these auto-regressive enhancements, thereby enriching the analytical toolkit available for such data types. As an example, we could imagine that one’s response to a visual analog scale item could create a temporary anchor point for the next, therefore influencing item responses over and beyond the latent causal attribute.

As we illustrated in this dataset, both the item response functions and the item information functions in the autoregressive IRT models are conditional upon succeeding the previous item(s). A first problem here is that the test information function of autoregressive IRT models is conditional upon the responses to each item that has a sequential effect on another. This prevents the typical examination of test information functions, where test information is seen as conditional upon person locations, but not conditional to all response patterns. Further, the implications of this remain to be discussed for tests that have multiple (or random) item presentation orders. Indeed, autoregressive models imply that, for the same set of items, the order in which the items were presented (and failed or succeeded) is important for the determination of ability. For example, person A succeeding an item $i_{1}$ and then failing a second item $i_{2}$, would, in a AR1-2PL, have a different estimated ability than person B, who would have failed $i_{2}$ and then succeeded item $i_{1}$. The resulting dependency of ability estimates on the order of item presentation has further consequences for computer adaptive tests, which usually select the next item to present based on the expected information provided by the next item. This expected information would then be conditional upon the order of presentation of the previous items, and upon whether the last item(s) presented were succeeded or not.

In spite of a better fit, we shall also note that some of the differences observed between the AR1-2PL and the 2PL models may be seen as relatively minimal. Notably, while the results suggest that reliability may be overestimated when not accounting for sequential effects, differences between person estimates were minimal. Therefore, it remains unclear whether an autoregressive approach is practically advantageous. Concerning the fact that the differences observed between the models were largely observed as differences in item difficulty levels and item discriminations but not person estimates, it may be that the autoregressive approach is particularly useful in situations where items are selected based on their parameters, such as computerized adaptive testing or optimal test assembly.

Finally, as we pointed out, a few studies have previously discussed how auto-regressive effects may be included in measurement models, notably with threshold auto-regressive IRT models for personality tests (Tang et al. 2020). The 2PL models may be seen as particular cases of these models. Therefore, the originality of the present research is not exactly the development of a novel approach. Instead, our goal was to highlight that local dependencies may arise from sequential effects in cognitive ability testing and in performance tests in general, as well as that IRT models can be extended to account for and/or study them efficiently. Failing to do so in our illustrative dataset led to a worse fit and an overestimated test reliability, and this could be the case in other contexts—which remain to be studied.

## Figures and Tables

**Table 1:** Item parameter estimates of the 2PL and AR1-2PL model (Mplus slope-intercept parametrization).

Model	Item	$\alpha_{j}$ (Discrimination)	SE	$\tau_{j}$ (Difficulty)	SE
2PL	1	0.855	0.146	−1.322	0.129
	2	1.997	0.324	−3.554	0.375
	3	1.692	0.232	−2.068	0.208
	4	4.089	0.730	−4.106	0.647
	5	4.933	1.018	−5.489	1.010
	6	2.375	0.317	−2.129	0.253
	7	1.550	0.198	−1.230	0.155
	8	1.612	0.199	−0.502	0.136
	9	1.264	0.163	−0.402	0.120
	10	2.196	0.286	0.703	0.166
	11	1.513	0.186	0.816	0.137
	12	1.136	0.157	0.910	0.125
AR1-2PL	1	0.856	0.150	−1.322	0.129
	2	1.816	0.318	−3.051	0.375
	3	1.502	0.228	−1.464	0.228
	4	3.950	0.833	−3.631	0.731
	5	4.187	0.936	−4.490	0.938
	6	2.019	0.311	−1.465	0.264
	7	1.243	0.197	−0.715	0.172
	8	1.379	0.199	−0.063	0.153
	9	0.971	0.165	−0.043	0.131
	10	2.016	0.307	1.045	0.177
	11	1.173	0.192	1.013	0.133
	12	0.911	0.159	1.097	0.126

## Data Availability

The data analyzed in this study is from (Myszkowski and Storme 2018), and is available at https://doi.org/10.1016/j.intell.2018.03.010.

## Abbreviations

The following abbreviations are used in this manuscript:

AIC	Akaike information criterion
AR	Auto-regressive
BCI	Bootstrapped confidence interval
BIC	Bayesian information criterion
CTT	Classical test theory
GLMM	Generalized Linear Multilevel Modeling
IRT	Item response theory
1PL	One-parameter logistic
2PL	Two-parameter logistic
3PL	Three-parameter logistic
4PL	Four-parameter logistic
RMSD	Root mean squared difference
SE	Standard error
SEM	Structural equation modeling
TAR	Threshold auto-regressive
